# Effects of curcumin in pediatric epithelial liver tumors: inhibition of tumor growth and alpha-fetoprotein *in vitro* and *in vivo* involving the NFkappaB- and the beta-catenin pathways

**DOI:** 10.18632/oncotarget.5673

**Published:** 2015-10-19

**Authors:** Nicola Bortel, Sorin Armeanu-Ebinger, Evi Schmid, Bettina Kirchner, Jan Frank, Alexa Kocher, Christina Schiborr, Steven Warmann, Jörg Fuchs, Verena Ellerkamp

**Affiliations:** ^1^ Department of Pediatric Surgery and Pediatric Urology, University Hospital Tuebingen, D-72076 Tuebingen, Germany; ^2^ Institute of Biological Chemistry and Nutrition, University of Hohenheim, Division of Biofunctionality and Safety of Food, D-70599 Stuttgart, Germany

**Keywords:** curcumin, hepatocellular carcinoma, cisplatin, pediatric, orthotopic tumor model

## Abstract

In children with hepatocellular carcinoma (pHCC) the 5-year overall survival rate is poor. Effects of cytostatic therapies such as cisplatin and doxorubicin are limited due to chemoresistance and tumor relapse. In adult HCC, several antitumor properties are described for the use of curcumin. Curcumin is one of the best-investigated phytochemicals in complementary oncology without relevant side effects. Its use is limited by low bioavailability. Little is known about the influence of curcumin on pediatric epithelial hepatic malignancies. We investigated the effects of curcumin in combination with cisplatin on two pediatric epithelial liver tumor cell lines. As mechanisms of action inhibition of NFkappaB, beta-catenin, and decrease of cyclin D were identified. Using a mouse xenograft model we could show a significant decrease of alpha-fetoprotein after combination therapy of oral micellar curcumin and cisplatin. Significant concentrations of curcuminoids were found in blood samples, organ lysates, and tumor tissue after oral micellar curcumin administration. Micellar curcumin in combination with cisplatin can be a promising strategy for treatment of pediatric HCC.

## INTRODUCTION

Malignant liver tumors in childhood are rare, the annual incidence rate for the United States was stated with 1.8 cases per million children younger than 15 years [1]. While hepatoblastomas (HB) account for the majority (91%) of these tumors, pediatric hepatocellular carcinoma (pHCC) accounts for approximately 1%. The outcome in hepatoblastoma has improved during the last decades, but still the patients with PRETEXT 4 stages suffer from poor 5-year-survival rates of 20–30% [2, 3]. The most cases of pediatric HCC present in advanced stages with poor outcome and 5-year overall survival rates of only 10–23% [1, 4]. Standard therapy consists in neoadjuvant PLADO therapy (Cisplatin – CDDP, and doxorubicin – DOXO), and resection of the tumors, respectively liver transplantation [5].

Recent attempts to improve the therapeutic options for patients with high risk or relapsing tumors include investigations of natural or synthetic chemopreventive agents. Curcumin, the predominant curcuminoid extracted from the rhizome of *Curcuma longa* Linn., is a phytochemical used in complementary oncology. With its pleiotropic effects on cellular signaling pathways, it decreases cancer cell proliferation and induces apoptosis [6]. In adult HCC, chemopreventive activities have been described, e.g. the amelioration of doxorubicin-associated cardiomyopathy and hypoxia-mediated sorafenib resistance [7, 8]. Moreover, curcumin inhibits diethylnitrosamine induced hepatocellular carcinoma in rats, and leads to apoptosis of HCC cells *in vitro* [9, 10].

Curcumin is known for its poor oral bioavailability. Incorporation of curcumin into micelles leads to an up to 185-fold enhanced bioavailability in healthy humans without causing adverse effects [11]. In children with inflammatory bowel disease it revealed an excellent tolerability of high doses (4g per day), and induced no side effects [12]. Despite its reported safety, there are currently no published studies describing the effects of curcumin on malignant epithelial pediatric liver tumors. We therefore aimed to investigate the therapeutic potential of native and highly bioavailable micellar curcumin [11] alone and in combination with cisplatin in pHCC. The established pediatric epithelial liver tumor cell lines HC-AFW1 and HepG2 [13, 14] were used in combination with an orthotopic pHCC mouse model.

## RESULTS

### Curcumin reduces viability of hepatocellular carcinoma cells

**Figure 1:**
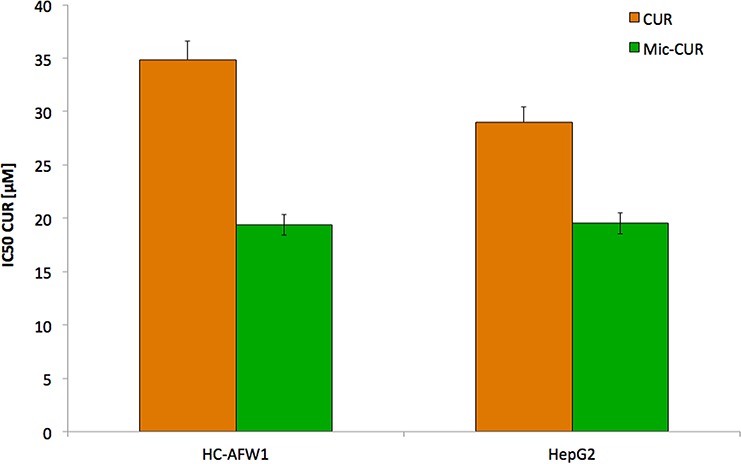
Native and micellar curcumin decrease viability of pHCC cells dose-dependently Incubation of HC-AFW1 cells and HepG2 cells with native and micellar curcumin for 72 hours; graph show IC50 of MTT test. Administration to native curcumin results in higher IC50. Differences were not statistically significant.

We initially compared the effects of native and micellar curcumin as well as unloaded micelles on the cell lines. There was no effect on fibroblasts, or of unloaded micelles on the cells (data not shown). Furthermore, native and micellar curcumin decreased the cell viability of both cell lines. In HC-AFW1, the following IC50 were determined: native curcumin, 34.86 μmol/L (CI95% 31.65–39.01); micellar curcumin, 19.38 μmol/L (CI95% 15.04–22.54). In HepG2, the IC50 for native curcumin was 29.07 μmol/L (CI95% 23.77–32.45) and for micellar curcumin 19.52 μmol/L (CI95% 15.31–21.98). The IC50 values for native curcumin were numerically higher than for micellar curcumin, but did not reach statistical significance (Figure 1). Native curcumin was therefore used for further *in vitro* experiments.

**Figure 2:**
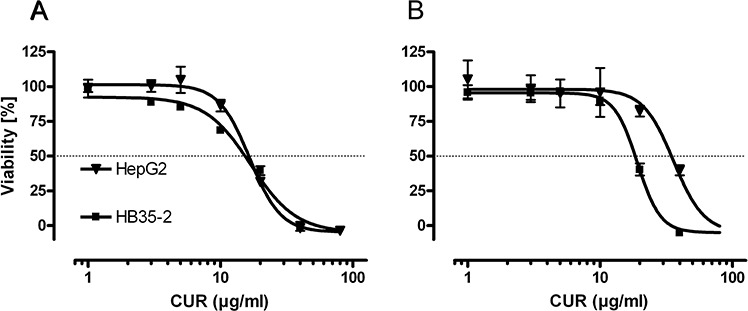
In high and low density pHCC cell cultures curcumin decreases cell viability Cell viability of HC-AFW1, and HepG2 cells in low **A.** and high **B.** density cell cultures after 48 h curcumin (CUR) administration. IC50 were lower in low density cultures (HC-AFW1: 16.95 (46.01 μM), CI95% 14.19–20.25; HepG2: 16.64 (45.17 μM), CI95% 15.39–17.98) compared to high density cultures (HC-AFW1: 19.17 (52.04 μM), CI95% 18.25–20.13; HepG2: 35.86 (97.35 μM), CI95% 29.76–43.21), however this was not significant.

**Figure 3:**
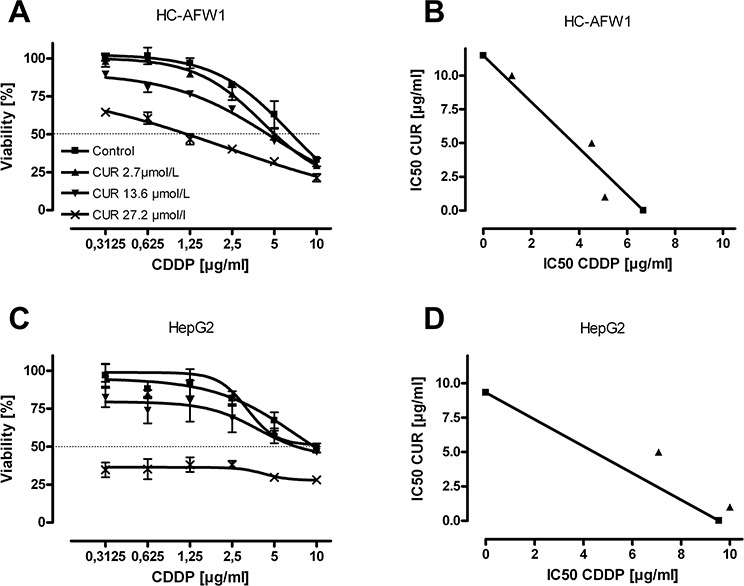
Combination of curcumin with CDDP acts additively on pHCC cells HC-AFW1 cells **A.** and HepG2 cells **B.** were cultured with increasing concentrations of CDDP and curcumin for 48 hours. Isobolograms show additive effects of combination therapy.

Even in high-density cell cultures, curcumin significantly decreased the cell viability. The IC50 for native curcumin determined in low-density cell culture experiments were 46.01 μmol/L (CI95% 38.52–54.97) in HC-AFW1 and 45.17 μmol/L (CI95% 41.78–48.81) in HepG2 cells. The native curcumin IC50 in high-density culture were 52.04 μmol/L (CI95% 49.54–54.64) for HC-AFW1 and 97.35 μmol/L (CI95% 80.79–117.29) for HepG2 cells (Figure 2). In combination with CDDP, curcumin operates additively on cell viability (Figure 3). The IC50 of CDDP decreased significantly under increasing curcumin concentrations: without curcumin the IC50 of CDDP was 6.68 μg/ml; after addition of 2.7 μmol/L the IC50 was 5.07 μg/L, after addition of 13.6 μmol/L the IC50 was 4.52 μg/L, and after addition of 27.2 μmol/L the IC50 was 1.19 μg/L.

**Figure 4:**
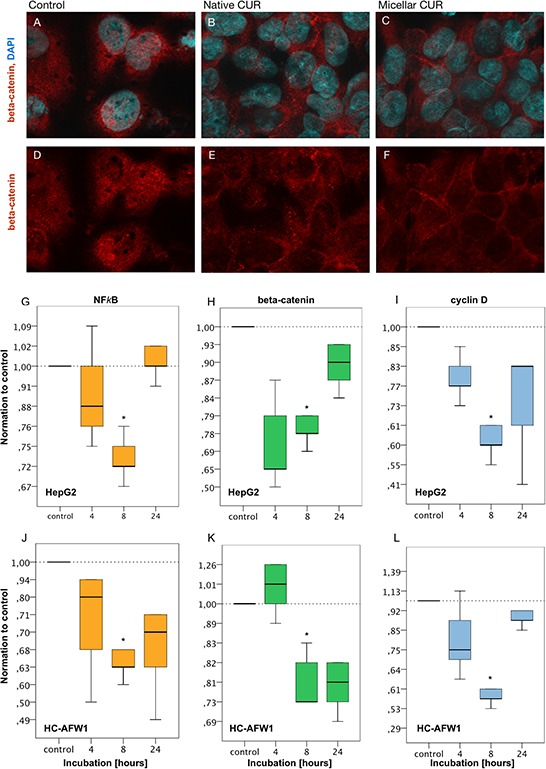
Curcumin modulates the distributional pattern of beta-catenin in HC-AFW1 cells in immunohistochemistry and reduces mRNA of beta-catenin, NFkappaB and cyclin D1 in RT-PCR Immunhistochemistry: HC-AFW1-were cultivated on slides (native, or micellar curcumin, 27.5μM, 24 hours). Confocal microscopy showed nuclear beta-catenin in untreated cells in contrast to cytoplasmatic and membranous beta-catenin after treatment with native or micellar curcumin. (upper row, **A–C.** nuclear counterstaining with DAPI, blue; beta-catenin immunostaining, red; x100). RT-PCR revealed significant decrease of mRNA of beta-catenin, NFkappaB and cyclin **D1.** after 8 hours curcumin incubation. **G–I.** HepG1 cells, curcumin 13.7 μmol/L; J-L: HC-AFW1 cells, curcumin 27.5 μmol/L.

In further studies we recently showed an inhibitory influence of beta-catenin inhibitors on hepatoblastoma cells, modulating the nuclear localization of beta-catenin [15]. In HC-AFW1 cells, beta-catenin is located mainly in the nucleus and plays an important role in cell proliferation [16]. Upon incubation of cells with low concentrations of native or micellar curcumin (1.8 μg/mL) for 24 h, a shift from nuclear beta-catenin towards cytoplasmatic and membranous beta-catenin was observed by confocal microscopy (Figure 4).

With real time PCR further analyses were carried out. Not only the expression of beta-catenin but also the expression of NFkappaB and cyclin D decreased significantly after 8 hours of incubation with curcumin. In HC-AFW1 cells, curcumin dosage was doubled compared to HepG2 cells (Figure 4).

### Micellar curcumin modulates tumor growth and decreases AFP levels

**Figure 5:**
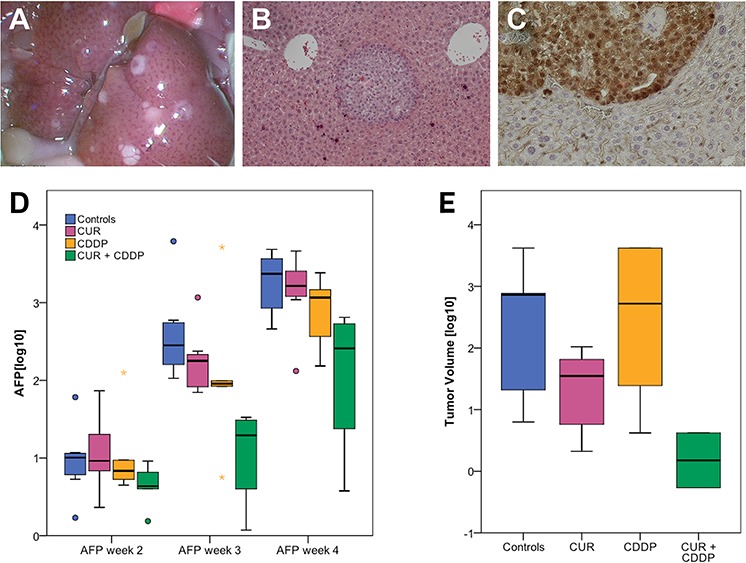
Tumor uptake in the orthotopic HCC model and AFP decrease after oral micellar curcumin feeding **A.** Tumor-uptake was 91.5% with multiple tumor nodules in the liver. **B.** H&E staining shows nodular intrahepatic growth of HC-AFW1-tumor (x 20). **C.** Immunohistological staining for beta-catenin revealed no change of the intranuclear distributional pattern after oral curcumin feeding. **D.** During therapy with CUR + CDDP, AFP values are significantly lower compared to controls (*). **E.** Tumor volumes are lowest after combination treatment. However, this reaches no statistical significance.

The tumor-uptake after intrasplenic tumor cell injection of HC-AFW1 cells into NSG mice was 91.5% (43/47) and the mice developed multiple intrahepatic tumor nodules (Figure 5A) with typical histology (Figure 5B). In contrast to the *in vitro* results, there was no difference between the beta-catenin distributional pattern between the groups in immunhistology (Figure 5C). AFP concentrations differed significantly between groups at week 3 (*p* = 0.006) and 4 ( *p* = 0.023), but not week 2 ( *p* = 0.35). The combination therapy (micellar curcumin + CDDP) significantly reduced AFP concentrations compared to control group (week 3: 1.04 ± 0.67 vs. 2.73 ± 0.64, *p* = 0.004; week 4: 2.05 ± 1.01 vs. 3.35 ± 0.43, respectively, *p* = 0.02). Compared to controls, AFP concentrations were numerically lower in mice treated with curcumin or CDDP individually, but this did not reach statistical significance (Figure 5D).

Overall, tumor volumes showed high variances. In the group of combination-treatment with curcumin + CDDP tumor volumes were lower than in the other groups; however, this did not reach statistical significance due to high variances (*p* = 0.075, Figure 5E).

**Figure 6:**
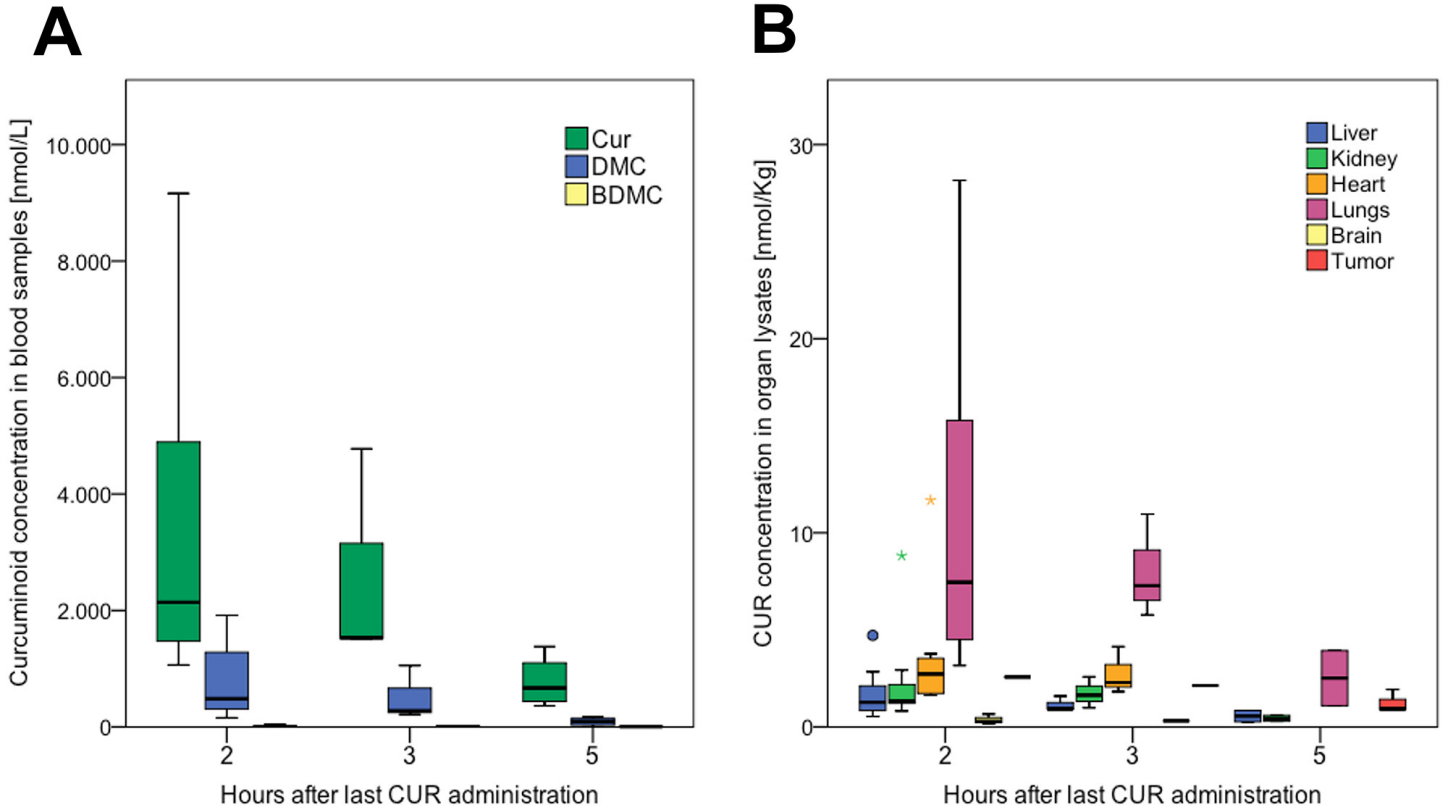
Bioavailability of curcumin in serum and organs after oral administration in mice **A.** Decrease of curcumin (CUR), demethoxycurcumin (DMC), and bis-demethoxycurcumin (BDMC) with time. After data normalization (log10), ANOVA revealed significant differences between the groups (*p* = 0.017); post-hoc test (Bonferroni) revealed significance between 2, respectively 5 hours (*p* = 0.16). **B.** Cur concentrations in organ lysates. After data normalization (log10), ANOVA revealed a significant difference between the groups (*p* = 0.000); post-hoc test (Bonferroni) revealed significantly higher concentrations in lungs compared to all other organs but heart, and significantly lower concentrations in the brain compared to all other organs but liver.

Oral administration of daily micellar curcumin did not cause any side effects, such as weight loss, diarrhea, or apathy, in the mice. In all analyzed blood samples from mice treated with curcumin alone or in combination with CDDP, significant concentrations of curcumin, DMC, and BDMC were found. Curcumin concentrations significantly differed between organs (*p* = 0.000) and the highest concentrations were observed in the lung and the lowest in the brain. The concentrations in the tumor tissue were higher than in the liver. The curcuminoid concentrations were highest 2 hours after administration and declined thereafter, showing that micellar curcumin is rapidly absorbed and reaches its maximum concentrations in less than 2 hours (Figure 6).

## DISCUSSION

HCC in children is extremely rare with an overall age-adjusted rate of 0.41 per 1,000,000 in children younger than 20 years [17]. The overall long-term survival in pHCC, however, is poor due to advanced disease at diagnosis and resistance to common drugs [1, 4]. In contrast to HCC in adults, the majority of pHCC cases are not related to hepatic cirrhosis [5]. Moreover, there are some biological differences on the molecular level between adult and pediatric HCC [18]. Thus, the transfer of results from basic research and treatment concepts from adult HCC to pHCC is limited.

In the present study, we assessed the therapeutic potential of curcumin on pHCC *in vitro* and *in vivo* on the basis of our newly described pHCC cell line HC-AFW1 [13]. We successfully established an orthotopic pHCC model in NSG mice with an excellent tumor uptake of 91.5%. Most authors describe models, in which either subcutaneously grown HCC tissue from one mouse is transplanted into the liver of another mouse [19] or HCC cells are injected directly into the liver [20]. Both methods bear the risk of uncontrollable bleeding; and in the tissue transplant model, the number of required laboratory animals is large because of the need for a donor and a recipient animal. The infrequent occurrence of liver metastases from a primary subcutaneously implanted tumor comes along with infrequent and unpredictable orthotopic growth [21]. Only few reports present data of tumor uptake-rates [22, 23]. We achieved an intrahepatic multinodular tumor growth through intrasplenic tumor cell injection. To avoid large tumors in the spleen, a splenectomy was carried out during the same anesthesia. Tumor growth was monitored by AFP detection in the serum as a surrogate marker of relative tumor burden and response to therapy, which is in line with our previous report of a high AFP expression in HC-AFW1-cells [13].

This orthotopic pHCC model served as the bases for further analyses of the effects of curcumin on HCC. Several studies provided evidence for a therapeutic potential of curcumin in the treatment of adult HCC [24]. A main barrier for the use of curcumin in clinical trials is its poor bioavailability and chemical instability [25]. Hence, several attempts to optimize the (oral) bioavailability of curcumin have been described. Only very few of the studies employing curcumin for cancer treatment in animals provided data on curcumin concentrations in serum, organs, or tumors [26–28]. In clinical trials, most of the tested galenics of curcumin achieve only low serum curcumin concentrations, if any [29–38], despite of using extremely high doses [29, 30, 39]. All these studies demonstrated, however, the safety of high doses of curcumin in humans. Only one micellar delivery system, which enhanced curcumin bioavailability in all tested subjects, was superior to all hitherto reported formulations [11]. This micellar curcumin was used in the *in vivo* experiments in this study. The micellar vehicle is a surfactant-based system to enhance the bioavailability for poorly soluble compounds for oral administration. However, the underlying mechanisms governing absorption from lipid and surfactant based formulations are not yet fully understood; however, release of compound is believed to take place either by partitioning from the intact vehicle, also referred to as interfacial transfer, or by degradation of the vehicle driving the compound out. The micelles theirselves do not enter the circulation [40]; their main role is a safe transport of the drug to the intestine, where the micelles are emulgated and then shattered while the drug itself is released and adsorbed by the cells of the small intestine. The administered oral dose of 60 mg/kg bodyweight (BW) was lower than in other reports (500 mg/kg BW, respectively 1000 mg/kg BW ) [27, 41]. The resulting serum concentrations of curcumin were high, but decreased rapidly with time. Five hours after oral curcumin feeding the mean serum concentration was decreased to less than 25% of the 2-hour value. In the organs, curcumin concentrations were heterogeneous. The standard deviations of the measurements were high, but it is of interest that the curcumin content in the tumors was higher than in the liver samples. We could show that the micellar galenics have overcome the often-reported poor bioavailability of curcumin and relevant concentrations were obtained in liver tumor tissue, and lowest concentration in the brain. Severe side effects, however, were not observed.

To assess the effects of curcumin on hepatoma cells, the sensitivity of HC-AFW1 and HepG2 cells to native and micellar curcumin was determined *in vitro.* As expected, incubation with curcumin led to a decrease in cell viability in both cells lines in a dose- and time-dependent manner. The calculated IC50 values are in agreement with several literature reports on the effects of curcumin in adult HCC models [42, 43]. Further, we combined curcumin with CDDP. The combination of curcumin with CDDP resulted in strong additive effects on cell viability in both cell lines. Similar effects of this combination were described for other cancer cells, but not for hepatoma cells yet [44]. This is of particular importance, as a number of studies provided evidence for protective properties of curcumin against CDDP-induced neurotoxicity, ototoxicity and nephrotoxicity [45]. In HC-AFW1 cells, primary tumor and xenografts, beta-catenin is predominantly localized in the nuclei [13]. In this study, we observed a shift from nuclear beta-catenin towards cytoplasmic and membranous beta-catenin under curcumin administration in both cell lines *in vitro*. A recently published meta-analysis pointed out that accumulated nuclear and membranous beta-catenin in adult HCC is an independent factor for poor prognosis and deep invasion [46]. The significant increase of beta-catenin, NFkB, and cyclin D1 depicts one of the mechanisms curcumin works in hepatoma cells. Physiologically a large proportion of beta-catenin is part of a complex of proteins such as e-cadherin that constitute adherens junctions between cells. Excess of cytoplasmatic β-catenin is phosphorylated and proteasomal degraded. In the presence of Wnt, the Axin-mediated phosphorylation/degradation of β-catenin is disrupted, allowing β-catenin to accumulate in the nucleus where it acts as a transcriptional factor for Wnt responsive genes, such as Cyclin D1. NFkB also acts as a transcriptional co-factor, e.g. for cyclooxygenase-2 (COX-2). COX-2 also is a risk factor in carcinogenesis of HCC, further analyses on COX-2 amounts in our model are outstanding. [47].

Curcumin alone did not have any significant influence on AFP concentrations or tumor volume *in vivo*. In contrast to our findings, some studies described significant reduction of subcutaneous HCC after intraperitoneal curcumin treatment [48–50]. One study even described a significant reduction of the relative areas of orthotopic tumors [50]. But in these studies, treatment with curcumin started early after tumor cell injection without any control of tumor growth before treatment [48, 49, 50]. Furthermore, intraperitoneal injection of curcumin circumvents the gastrointestinal barrier and thus facilitates significantly higher tissue concentrations than following oral administration [26]. Tumor volume is an unreliable marker of tumor activity as even large tumors may consist of high amounts of necrotic areas. Additionally, in orthotopic models, tumor volume measurements are unreliable due to the multinodular growth and difficulty of exact recording. Although the diagnostic value of AFP is questioned, AFP measurement is still the standard diagnostic marker for HCC [51]. In literature, there is no data provided concerning AFP concentrations before and after treatment with curcumin [48–50]. Therefore, the curcumin effects in the above mentioned studies show a preventive effect of curcumin rather than a therapeutic effect. In our model, the combination of curcumin with CDDP treatment significantly decreased AFP concentrations. Tumor volumes were also lower under combination treatment, but due to high variance, this was not significant. In a model of head and neck cancer a significant reduction of tumor volume was described after combination of CDDP with a curcumin analogue (H-4073), but also after H-4073 alone [52].

In conclusion, oral administered micellar curcumin has a good bioavailability and enriches in hepatocellular tumor tissue. The decrease of AFP levels in tumor bearing mice is promising for the use of additive micellar curcumin in the treatment of children with HCC. Possible ways of action in hepatocellular carcinoma cells are the inhibition of the NFkB-, and the Wnt/beta-catenin-pathway, resulting in cyclin D1 decrease.

## MATERIALS AND METHODS

### Drugs and phytochemicals

The native curcumin powder (Jupiter Leys, Cochin, Kerala State, India) used in all formulations contained 82% curcumin, 16% demethoxycurcumin (DMC), and 2% bis-demethoxycurcumin (BDMC). Curcumin micelles were composed of 7% curcumin powder (equivalent to 6% curcumin) and 93% Tween-80 (Kolb, Hedingen, Switzerland) and were manufactured by AQUANOVA AG (Darmstadt, Germany) [11]. All percentages refer to weight. The cytotoxic agent cisplatin (CDDP) was used as drug formulation (Neocorp AG, Weilheim, Germany).

### Cells and cell culture

The cell line HepG2 (trabecular type, LGC Promochen, HB8065, Salisbury, United Kingdom) was initially declared as pHCC cell line, meanwhile, they are classified as hepatoblastoma cell line [53, 14]. The cell line HC-AFW-1 originates from a pHCC [13]. Cells were grown in Dulbecco's modified Eagle's medium (DMEM; Biochrom, Berlin, Germany), supplemented with 10% fetal bovine serum (FCS, Biochrom), 1% Penicillin/Streptomycin (Biochrom), and 1% L-glutamine (Biochrom). Cell plastic ware was purchased from Greiner, Essen, Germany. For *in vivo* experiments, cells were resuspended in sterile PBS without FCS and injected into mice (see *animal study*).

### Immunocytochemistry

For immunocytochemistry of beta-catenin, 3 × 10E4 cells were cultured on chamber slides (NUNC). For better cell adherence, slides were coated with Poly-D-lysine (Sigma-Aldrich). Each chamber was filled with 3 × 10E4 cells in 100 μl culture media and incubated overnight. After 24 hours incubation of 0.05 μM curcumin cells were 15 minutes fixed in acetone-methanol (1:1) at −20°C. Then cells were permeabilized with PBS supplemented with 2% Tween-20. The staining was performed with a polyclonal anti-rabbit beta-Catenin antibody (1:500, Cytomed). Cells were washed 3 times in PBS with 2% Tween-20 and incubated with anti-goat Cy3 (1:200) secondary antibody for 1 hour. Nuclei were stained with DAPI (1:2000). Then cells were mounted with mounting medium after 3 times of washing. Confocal-style three-dimensional imaging was performed using an Axio imager 2 microscope with ApoTome system (Carl Zeiss, Jena, Germany). Images were processed using AxioVision 4.8.1 software.

### Proliferation assay

HepG2 and HC-AFW were cultured in 96-well plates (Becton Dickinson GmbH, Heidelberg, Germany) at a low density (5.000 cells/100μl), and high density (25.000 cells/100 μl). Cells were treated with 6 different concentrations of CDDP around IC50 (0,3125–10 μg/mL) [54]. Experiments were repeated with fibroblasts and native curcumin, micellar curcumin, and unloaded micelles, as well as curcumin in combination with CDDP, respectively. Drugs solutions were prepared shortly before administration. Cell viability was determined after 48 h by colorimetric MTT [3-(4.5-dimethyl-thiazol-2-yl)-2.5-diphenyl-tetrazoliumbromide] assay (Sigma-Aldrich, Munich, Germany). The assay was performed as descripted before [15]. Dose-dependent viability curves were computed by sigmoidal curves with variable slope to determine IC50 using the GraphPad Prism Software. For further analysis, the relative viability was assessed and the IC50 values for each drug administered alone, or in combination with a fixed concentration of curcumin were established from the concentration-effect curves. The IC50 values of co-treatment were divided by the IC50 value of each drug in the absence of the other drug. In a graphical presentation, the straight line connecting the IC50 values of the two agents when applied alone corresponds to additivity, or independent effects of both agents. Values below this line indicate synergy; values above this line indicate antagonism [55]

### Real time PCR

To determine the mRNA abundance of the beta-catenin, cyclin D1 and NFkappaB, total cellular RNA was extracted from human pHCC cell lines HepG2 (trabecular type, LGC Promochen, HB8065, Salisbury, UnitedKingdom) and HC-AFW1 [11] using the RNeasy-Minikit (Qiagen, Hilden, Germany) according to the manufacturer's instructions. After DNAse digestion approximately 2.5 μg of total RNA was reverse transcribed to cDNA using High capacity cDNA reverse transcription kit (Life technologies, USA). Quantitative real-time PCR was applied on the CFX96 Real-Time System (Biorad) using 500 nM forward and reverse primer and 2x GoTaq^®^ qPCR Master Mix (Promega Corporation, Madison, WI, USA) according to the manufacturer's protocol. Cycling conditions were as follows: initial denaturation at 95°C for 5 minutes, followed by 40 cycles of 95°C for 15 seconds, 58°C for 30 seconds and 72°C for 30 seconds. For the amplification of human pHCC HepG2 and HC-AFW1 cells the following primers were used (5′-3′orientation):

Beta-catenin, fw; GCCCGAAACGCCGAATAT, and rev; CCGTGGTTCGTGGCTGCTCTC
Cyclin D1, fw; CCGTCCATGCGGAAGATC, and rev; ATGGCCAGCGGGAAGAC
NFkappa B, fw; CGAGACAGTGACAGTGTCTGC, and rev; GCTCTCTGAGCACCTTTGGATG.

The transcript level of TATA box binding protein (TBP) as housekeeping gene was determined for each sample using the following primers (5′ → 3′ orientation):

TBP, fw GCC CGA AAC GCC GAA TAT, and rev CCG TGG TTC GTG GCT CTC.

Specificity of PCR product was confirmed by analysis of a melting curve. All experiments were done in duplicate. Relative quantification of gene expression was achieved using the Δct method.

### Mice

NOD/LtSz-scid/IL-2Rgamma(null) mice (NSG mice) were purchased from Charles River (Sulzfeld, Germany) and bred in our facility. Sterilized food and water were accessible ad libitum. For inclusion in the animal study, a minimum body weight of 20 g and a minimum age of 6 weeks were required. Both, male and female mice were used with systematic allocation to the experimental groups. The animal study to assess tumor establishment and treatment efficacy was in accordance with the Guide for the Care and Use of Laboratory Animals (NIH publication 86–23, revised 1985) and in compliance with local regulations and approved by the responsible authority (Regierungspräsidium Tübingen, K 6/13).

### Animal study

For induction of intrahepatic pHCC tumor growth, 47 mice received 2 × 10^6^ HC-AFW-1 cells into the spleen before splenectomy, similar to the recently described orthotopic hepatoblastoma model [56]. By the increase of serum alpha fetoprotein (AFP) > 5 U/mL, mice were randomly assigned to one of four groups: control (*n* = 13; no treatment); curcumin (*n* = 10; daily administration of micellar curcumin by pipetting a glucose-micellar-curcumin-solution, 60 mg/Kg body weight, 5 days a week for three weeks); CDDP (*n* = 10; intraperitoneal injections of 1 mg/kg bodyweight on days 1 and 2), and curcumin + CDDP (*n* = 10; combination therapy consisting of intraperitoneal injections of CDDP on days 1 and 2 and oral gavage of curcumin micelles on 5 days a week for three weeks).

### Tumor monitoring

Mice were visited daily and studied for any abnormal behavior or wound infections. Blood samples were taken weekly from the retro-orbital plexus of CO_2_/O_2_-anaesthetized mice. The tumor marker human AFP was measured weekly in serum using an ELISA Kit (DRG Instruments, Marburg, Germany) and expressed in IU/mL. Therapy started when AFP serum concentrations were > 5 U/mL. Three weeks after the beginning of therapy, mice were sacrificed by cervical dislocation in CO2 anesthesia. Intraperitoneal organs, lungs, and brains were exposed and macroscopically assessed for existence of tumors. Tumors were counted and each tumor diameter (d) measured. The volume of each tumor nodule per mouse was calculated by the formula volume = 1/6 πd^3^ and then summated. A portion of each tumor was fixed in 4% buffered formaldehyde and processed for histological analysis. Other organs were snap frozen in liquid nitrogen and stored at −80°C.

### Blood sampling and processing

For the determination of plasma curcuminoid concentrations (curcumin, bis-demethoxycurcumin (BDMC), and demethoxycurcumin (DMC)), blood was collected from mice of the curcumin and curcumin + CPPD groups 2, 3, and 5 h after the last curcumin administration. Different mice were used for each time point. Blood was collected in EDTA tubes (Sarstedt AG & Co, Nümbrecht, Germany) and immediately centrifuged (1008 × g, 10 min, 4°C). The obtained plasma samples were stored at −80°C until further analysis.

### Organ lysates and processing

Thawed mouse brain, liver, tumor, kidney, lung, and heart was weighed into 2 mL tubes and homogenized in 200 μL 0.1 M sodium acetate buffer (pH 4.0–4.5) containing 1.6% EDTA (48.8 μmol/L) and 2.5% ascorbic acid (25 μmol/L) using a Miccra D-1 homogeniser (ART Prozess- & Labortechnik GmbH & Co. KG, Müllheim, Germany) at 18,000 rpm. Curcumin was extracted and reconstituted as described below.

### Curcumin detection in plasma and organ lysates

Curcumin, BDMC, and DMC in plasma and tissues were extracted using a modified method of Heath et al., as described elsewhere [11]. One-hundred μL of plasma or tissue sample homogenates were incubated with 1000 U β-glucuronidase (from Helix pomatia, Sigma, St. Louis, USA) dissolved in 0.1 M sodium acetate buffer (pH 4.0–4.5) at 37°C under agitation. Afterwards, 1 mL extraction solvent (95% ethyl acetate, 5% methanol, Carl Roth GmbH+Co.KG, Karlsruhe, Germany) was added and vortex-mixed. Subsequently, samples were centrifuged (10,500 × g, 5 min, 4°C) and supernatants collected. This step was repeated twice. The organic layer was evaporated to dryness using an RVC 2–25 CDplus centrifugal evaporator (Martin Christ Gefriertrocknungsanlagen GmbH, Osterode am Harz, Germany). Samples were re-suspended in 150 μL methanol, vortex-mixed, left in the dark for 10 min, and vortex-mixed again, and then transferred to an HPLC vial. Curcuminoids were quantified on a Jasco HPLC system (Jasco GmbH, Gross-Umstadt, Germany) with a fluorescence detector (excitation wavelength 426 nm, emission wavelength 536 nm) and separated on a Reprosil-Pur C18-AQ column (150 mm × 4 mm, 3 μm particle size; Dr. Maisch GmbH, Ammerbuch, Germany) maintained at 40°C. The mobile phase consisted of 52% deionised water (adjusted to pH 3 with perchloric acid), 34% acetonitrile, and 14% methanol and was delivered at a flow rate of 1.4 mL/min. Curcuminoids were quantified against external standard curves (curcumin, purity ≥ 97.2%, CAS # 458–37-7; DMC, purity ≥ 98.3%, CAS # 22608–11-13; BDMC, purity ≥ 99.4%, CAS # 24949–16-0; Chromadex, Irvine, USA).

### Statistics

For statistical analyses, SPSS (Version 22.0) was used. Decision for parametric or non-parametric tests was made after Shapiro-Wilk testing for low numbers of data. To achieve normal distribution, data were transformed to the base-10 logarithm. In case of normal distribution, data are given as means and 95% confidence intervals (95% CI). Comparisons of AFP values and curcumin concentrations were performed by one-way analyses of variance (ANOVA) with Bonferroni posthoc test. A *p*-value of 5% or lower was considered to be statistically significant. Statistical uncertainty was expressed as Clopper-Pearson 95% confidence interval (CI95%).
